# Prediction of Severe Esophageal Varices in Patients With Cirrhosis Based on Levitt’s CO Breath Test: A Proof of Concept Study

**DOI:** 10.1097/MCG.0000000000001768

**Published:** 2022-09-30

**Authors:** Chu-Wu Feng, Ling-Ling Kang, Hou-De Zhang

**Affiliations:** * Department of Critical Care Medicine, People’s Hospital of Longhua; † Department of Gastroenterology, Nanshan Hospital, Guangdong Medical University, Shenzhen, China

**Keywords:** RBC lifespan, Levitt’s CO breath test, varices, cirrhosis

## Abstract

This study investigated the feasibility of using erythrocyte (RBC) lifespan determined by Levitt’s CO breath test (LCOBT) to predict esophageal varices needing treatment (VNT) in patients with cirrhosis.

**Background:**

Esophageal varix bleeding is a common fatal complication of cirrhosis and portal hypertension. The gold standard for identifying VNT is esophagogastroduodenoscopy (EGD), an invasive procedure with low patient compliance. VNT screening based on Baveno VI criteria has mediocre specificity.

**Study:**

RBC lifespan was determined by LCOBT in 53 cirrhotic patients (13 without varices, 11 mild/moderate varices, and 29 severe varices). Correlation of varix severity with RBC lifespan and other variables was analyzed. Rates of shortened RBC lifespan and thrombocytopenia (Baveno VI criteria) were compared.

**Results:**

RBC lifespan correlated inversely with severity of varices (*r*=−0.793, *P*<0.001). Mean RBC lifespans were 129±31, 96±21, and 59±21 days for Nonvarix, Mild/Moderate, and Severe groups. Shortened RBC lifespan (<75 d) was observed in 79.3% (23/29) of patients with severe varices, a frequency similar or identical to thrombocytopenia rates [original Baveno VI criteria, 86.2% (25/29), *P*=0.487; expanded criteria, 79.3% (23/29), *P*>0.999]. Among 24 patients without severe varices, shortened RBC lifespan was observed in 1 patient whereas thrombocytopenia was detected in 13 and 8 patients based on the original (*P*<0.001) and expanded criteria (*P*=0.010), respectively.

**Conclusions:**

RBC lifespan correlates inversely with varix severity in patients with cirrhosis. LCOBT may enable specific screening for VNT.

Esophageal varix bleeding is a common and potentially fatal complication of cirrhosis and portal hypertension, with a 20% mortality rate within 6 weeks of hemorrhage onset.^1^ The identification of varices needing treatment (VNT) due to their severity and associated high rupture risk is critical for minimizing serious varix hemorrhage and consequent mortality. Esophagogastroduodenoscopy (EGD) is the gold standard method for determining varix severity and thus identifying VNT.^2^

Typically, EGD is performed in cirrhotic patients at the time of diagnosis, and then endoscopic follow-up is indicated every 1 to 2 years thereafter in patients with small varices and every 2 to 3 years thereafter in patients with no visible varices.^2^ However, patient compliance with prescribed EGD is very low, likely due to the invasive nature of it. In a survey of 423 patients diagnosed with cirrhosis in the United States, only a third of the surveyed patients completed their recommended EGD screening for esophageal varices in accordance with the guidelines.^3^ In China, the situation is particularly troubling because in addition to poor compliance by patients, there is also low physician awareness of EGD screening.^4^ The wide availability of noninvasive diagnostic methods is now enabling cirrhosis to be diagnosed earlier than in previous decades during a stage of effective compensation, resulting in a greater frequency of futile EGD at initial screening appointments.^5^ Furthermore, futile follow-up endoscopies are also expected to increase in frequency consequent to the availability of effective antiviral agents that can cure hepatitis infection, and thus reduce the risk of hepatitis-associated cirrhosis and the hepatitis sequelae of portal hypertension and varices.^6^

Noninvasive methods for esophageal varix screening and surveillance that may reduce the need for EGD procedures in patients with cirrhosis are being tested intensively, including biochemical marker assays, imaging techniques, and diagnostic models. Unfortunately, their performance and feasibility remain, as of yet, controversial.^7^ Currently, the only noninvasive varix screening and surveillance procedure that is recommended is the application of the Baveno VI criteria, which use transient elastography and platelet count values to determine varix risk (original version launched in 2015; expanded version proposed in 2017).^8,9^ Specifically, the criteria state that endoscopic screening for VNT can be obviated in patients with a liver stiffness below 20 kPa and a platelet count (PLT) above 150×10^9^/L or 110×10^9^/L (Expanded-Baveno VI criteria) at the time of diagnosis. Otherwise, according to the criteria, it would be prudent to conduct endoscopic screening. A 2019 meta-analysis and 2 further validation studies showed that the Baveno VI criteria had high sensitivity (90% to 97%) but only mediocre specificity (32% to 51%).^6,10,11^ Consequently, the total number of endoscopies avoided using the criteria is relatively low.^6,7^ Furthermore, transient elastography is not widely available in developing areas. Therefore, there remains a need for cost-effective, noninvasive screening, and surveillance techniques that provide a reliable and reproducible prediction of VNT in patients with cirrhosis.

In healthy adults, erythrocyte (RBC) lifespan (mean period of RBC survival in circulation after RBC release from bone marrow) is typically about 120 days.^12,13^ An abnormally short RBC lifespan is a fundamental characteristic of hemolysis. Portal hypertension can cause hypersplenism characterized by splenomegaly and accelerated destruction of RBCs (extravascular hemolysis), leukocytes, and platelets. Considering that peripheral blood PLT correlates with severity of esophageal varices in patients with portal hypertension,^8,9^ we became keen to determine whether a similar correlation may also exist between VNT risk and RBC lifespan.

RBC lifespan is seldom measured in basic research or clinical practice due to the cumbersome and protracted nature of classic ^51^Cr-labeling methods of RBC lifespan measurement, which can take several weeks or even months. On the basis of the knowledge that endogenous CO in exhaled air comes mainly from hemoglobin degradation after RBC destruction and that lungs are the body’s only route of CO excretion, Levitt’s research team developed a simple rapid CO breath test to determinate RBC lifespan that yields results that are similar to those obtained by classic standard methods.^14,15^

Patients with cirrhosis have increasing carboxyhemoglobin in their blood and exhale abnormally high levels of CO.^16–20^ Therefore, in this proof of concept study, we used Levitt’s CO breath test (LCOBT) to measure the RBC lifespan of the cirrhotic patients with esophageal varices of various severities, so as to explore the possibility to predict VNT risk reliably by the simple breath test.

## MATERIALS AND METHODS

### Subjects

The study sample included 53 patients with cirrhosis (36 men and 17 women, age range: 31 to 73 y) who underwent EGD for dyspepsia at Nanshan Hospital between July 2018 and May 2020. Cirrhosis diagnosis was based on patient history, physical examinations, laboratory tests, imaging findings, and (in a minority of cases) liver biopsy. The inclusion criteria were nonsmoker status and no history of esophageal variceal bleeding or invasive therapy for varices. The exclusion criteria were cigarette smoking; hepatocellular carcinoma; splenectomy; currently in menstrual phase for women, blood transfusion, nonvariceal bleeding, or use of a hemolytic drug (eg, ribavirin) within 1 week before breath testing; severe cardiopulmonary disease; acute disease; and inability to finish gas sample collection. The study protocol was approved by the Ethics Committees of Nanshan Hospital. All patients gave informed consent.

### EGD

EGD was performed in accordance with routine hospital procedures. Esophageal varix severity was classified according to the 2016 Chinese Guidelines^21^ as follows: Nonvarix, no varices visible in esophagus or stomach; Mild, varices present that run straight; Moderate, varices present that run snake-like/oblique; and Severe, presence of tortuous varices with a beaded or tumor-like appearance. Mild-class and Moderate-class varices with red coloring were reclassified as moderate and severe, respectively. Finally, the Mild and Moderate classifications were combined into a single Mild/Moderate group for our analyses.

### LCOBT

LCOBTs to determinate RBC lifespan were carried out within1week after endoscopy. The principle underlying the method is that the lungs are the body’s sole means of excreting CO and that endogenous CO in exhaled breath originates mainly (∼70%) from heme oxidation during hemoglobin degradation following RBC rupture, such that the total capacity of CO from hemoglobin divided by the CO quantity released per day equates to mean RBC lifespan.^14,15,22,23^

All LCOBTs were conducted in the morning (08:00–11:30) without a fasting requirement. Briefly, to collect an alveolar sample, each subject was instructed to expel their breath into a foil bag, filling it (700 ml). An equal-volume atmospheric air sample was collected immediately after the collection of the alveolar sample. The same day, a peripheral blood sample was taken to conduct complete blood counts for Hb used in RBC lifespan calculation in Levitt’s formula.

Breath testing was carried out with an automated instrument (ELS TESTER, Seekya Biotec Co. Ltd, Shenzhen) that determines alveolar endogenous CO concentration by nondispersive infrared spectroscopy of paired alveolar and air gas samples. Using that measurement and a Hb datum determined by the aforementioned blood test, the instrument determines RBC lifespan automatically with Levitt’s formula within 15 minutes. The mean normal RBC determined with this method has been shown to be 126 days (range, 75 to 177 d), which is similar to that obtained by the biotin-labeling technique (mean, 115 d; range, 70 to 140 d).^23^

### Statistical Analysis

Normally distributed data (age, Hb, RBC count, and RBC lifespan) are reported as means with standard deviations. Non-normally distributed data (WBC and PLT) are given as medians with interquartile ranges. Enumerated categorical data (gender, splenomegaly, Child-Pugh class, and hemocytopenia) are expressed as group percentages. Normally distributed, non-normally distributed, and enumerated categorical data were compared across varix severity groups with Student *t* tests or analyses of variance, Wilcoxon rank-sum tests, and χ^2^ tests, respectively. Correlations between esophageal varices with RBC lifespan and other variables of interest were determined by Spearman analyses. Partial correlation analysis was used to detect independent correlations among variables. The statistical analyses were conducted in SPSS v. 22.0 for Windows (SPSS, Chicago, IL). *P*<0.05 was considered significant.

## RESULTS

### Patient Characteristics

The study’s sample of 53 eligible patients with cirrhosis were divided into esophageal varix severity groups based on EGD findings as follows: Nonvarix, N=13; Mild/Moderate, N=11; and Severe, N=29. None of the patients in the Nonvarix group had gastric varices, in addition to having no esophageal varices. The groups’ baseline demographic and clinical characteristics are summarized in Table 1. The groups had similar gender and age compositions. More advanced progression of esophageal varices tended to show higher splenomegaly rates, more compromised liver function, and lower peripheral blood cell counts.

**TABLE 1:** Characteristics of Patient Groups Based on Varix Severity

	Esophageal varices		
Variable	Nonvarix (N=13)	Mild/moderate (N=11)	Severe (N=29)
Age, y	52±12	55±12	53±10
Gender			
Male	7 (53.8%)	7 (63.6%)	22 (75.9%)
Female	6 (46.2%)	4 (36.4%)	7 (24.1%)
Splenomegaly	2 (15.4%)	8 (72.7%)^**^	27 (93.1%)^**^
Child-Pugh class			
A	12 (92.3%)	9 (81.8%)	9 (31.0%)^**^ ^##^
B	1 (7.7%)	2 (18.2%)	16 (55.2%)
C	0	0	4 (13.8%)
WBC, 10^9^/L	5.3 (3.8, 6.0)	3.7 (3.1, 5.1)	3.3 (2.4, 5.9)
Leukocytopenia^†^	4 (30.8%)	6 (54.5%)	18 (62.1%)
RBC, 10^12^/L	4.19±0.72	4.08±0.67	3.65±0.59^*^
Hb, g/L	126±22	123±17	111±21
Anemia^‡^	3 (23.1%)	2 (27.3%)	16 (55.2%)^*^
PLT, 10^9^/L	167 (135, 194)	84 (71, 128)^**^	61 (44, 104)^**^
Thrombocytopenia			
<150×10^9^/L^§^	4 (30.8%)	9 (81.8%)^**^	25(86.2%)^**^
<110×10^9^/L^‖^	0	8 (72.7%)^**^	23 (79.3%)^**^

Data are reported as mean±SD, median (interquartile range), or N (%).

* *P*<0.05.

** *P*<0.01 versus Nonvarix.

## *P*<0.01 versus Mild/Moderate.

† WBC<4.0×10^9^/L.

‡ Hb<120 g/L for males and<110 g/L for females.

§ Baveno VI.

‖ Expanded-Baveno VI.

Hb indicates hemoglobin concentration; PLT, platelet concentration; RBC, erythrocyte; WBC, white blood cell.

### RBC Lifespan

**FIGURE 1:**
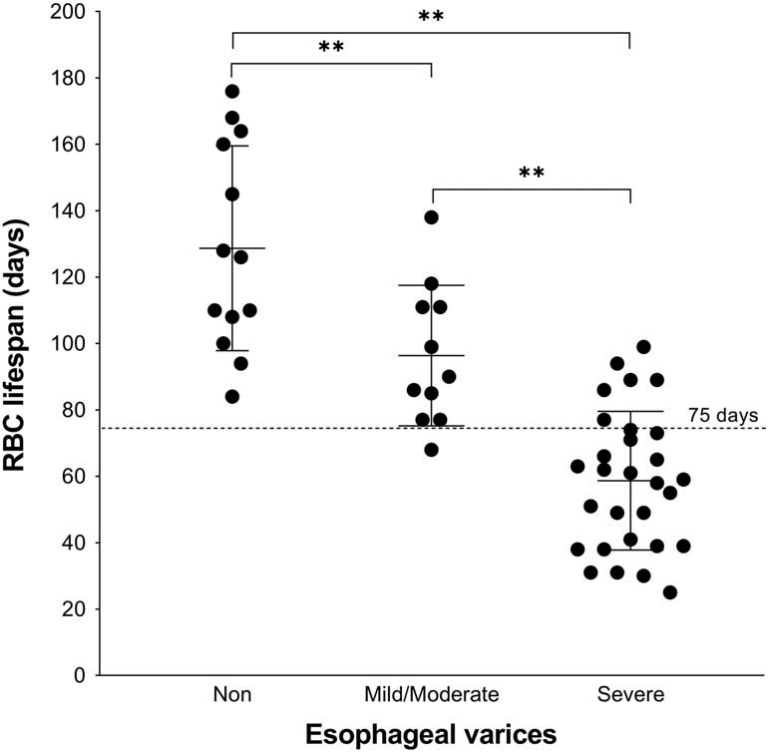
Scatter plot of RBC lifespan data obtained from 53 patients with cirrhosis. Each data point represents the RBC lifespan for1patients determined by LCOBT. The Severe group had shorter RBC lifespans than both the Mild/Moderate group and the No-varix group while the Mild/Moderate group had shorter RBC lifespans than the No-varix group; **P<0.01. LCOBT indicates Levitt’s CO breath test; RBC, erythrocyte.

The Severe group (mean±SD, 59±21 d; *N*=29) had shorter RBC lifespans than both the Mild/Moderate group (96±21 d; N=11) and the Nonvarix group (129±31 d; N=13), whereas the Mild/Moderate group had shorter RBC lifespans than the Nonvarix group (Fig. 1). The RBC lifespan in the Nonvarix group was similar to the normal reference value of 126 days (*t*=0.314, *P*=0.759).

### Factors Related to Severity of Esophageal Varices

**FIGURE 2:**
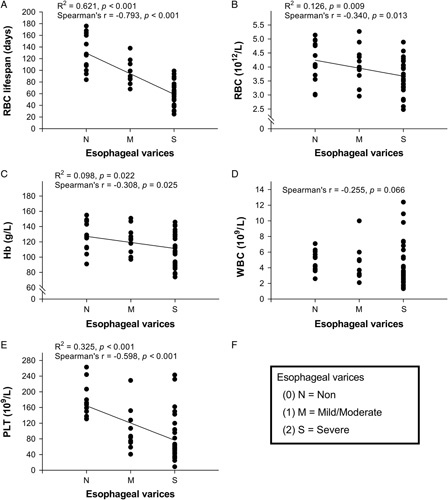
Plots of correlations of varix severity with RBC lifespan (A), complete blood counts (B–E). Varix severity groups are identified on the x-axes in accordance with the legend (F).

Bivariate correlation analyses examining trends in RBC lifespan and other variables (see Table 1) across varix severity groups revealed that RBC lifespan was the assessed variable that correlated most robustly with varix severity (*r*=−0.793, *P*<0.001) (Fig. 2), followed by splenomegaly (*r*=0.656, *P*<0.001), PLT (*r*=−0.598, *P*<0.001), Child-Pugh class (*r*=0.565, *P*<0.001), RBC (*r*=−0.340, *P*=0.013), and Hb (*r*=−0.308, *P*=0.025). Neither WBC (*r*=−0.255, *P*=0.066), gender (*r*=−0.199, *P*=0.152), nor age (*r*=0.029, *P*=0.837) correlated with severity. The correlation strength rankings of these variables remained the same after variable normalization and re-analysis (data not shown). Partial correlation analysis confirmed a negative correlation between varix severity and RBC lifespan (*r*=−0.552, *P*<0.001).

### Diagnostic Performance

An RBC lifespan shorter than the breath-test normal lower limit of 75 days was observed in 23/29 patients (79.3%) with severe esophageal varices, including 6/9 patients (66.6%) with Child-Pugh class A liver function, but only 1/24 patients (4.2%) without severe varices. This difference was confirmed to be consistent with a significant association between the presence of severe esophageal varices and RBC lifespan determined by LCOBT (χ^2^=29.927, *P*<0.001).

PLT is1of the 2 parameters in Baveno VI criteria and is available for patients subjected to LCOBT because such patients have a complete blood count panel performed to determine Hb for RBC lifespan calculation in Levitt’s formula. Therefore, we calculated thrombocytopenia rates for each group based on the patients’ PLT values and compared the results with the predicted VNT rate obtained based on abnormal RBC lifespan shortening. We found that the rate of abnormal short RBC lifespan in patients with severe esophageal varices was similar to the thrombocytopenia rate defined by the original Baveno VI criteria and was identical to the thrombocytopenia rate defined by the Expanded-Baveno VI criteria (Table 2). Among the patients without severe varices, there was only a single case in which the patient had an abnormally short RBC lifespan while more than half met the PLT cutoff for thrombocytopenia based on the original Baveno VI criteria and a third met the PLT cutoff for thrombocytopenia based on the Expanded-Baveno VI criteria (Table 2).

**TABLE 2:** Abnormally Short RBC Lifespan and Thrombocytopenia in Cirrhotic Patients With and Without Severe Esophageal Varices

Cutoff	Severe (N=29)	Mild/Moderate + Nonvarix (N=24)
RBC lifespan<75 d	23 (79.3)	1 (4.2)^##^
Thrombocytopenia		
PLT<150×10^9^/L^†^	25 (86.2)	13 (54.2)^#^ ^**^
PLT<110×10^9^/L^‡^	23 (79.3)	8 (33.3)^##^ ^*^

* *P*<0.05.

** *P*<0.01 versus RBC lifespan<75 days.

# *P*<0.05.

## *P*<0.01 versus Severe.

† Data are N (%). Baveno VI.

‡ Expanded-Baveno VI.

PLT indicates platelet concentration; RBC, erythrocyte.

## DISCUSSION

In the present study with 53 cirrhotic patients, an abnormally shortened RBC lifespan based on LCOBT measurements (<75 d) was observed in nearly four fifths of the 29 patients with severe esophageal varices but observed in only a single patient among the 24 without severe esophageal varices. The rate of abnormally short RBC lifespan in patients with severe esophageal varices was similar to and identical to the thrombocytopenia rates obtained with the original Baveno IV criteria and with the Extended-Baveno IV criteria, respectively. In patients without severe varices, the abnormally low RBC lifespan rate was dramatically lower than the thrombocytopenia rates obtained with these sets of criteria.

RBC lifespan shortening is characteristic of hemolysis, and extravascular hemolysis is the essential manifestation of hypersplenism, which is the most common major complication of portal hypertension due to various causes, including cirrhosis.^1^ Therefore, it is not surprising to find a shortened RBC lifespan in patients with cirrhosis. However, our finding of a robust correlation between esophageal varix severity and RBC lifespan shortening that was more robust than correlations with multiple other examined hypersplenism-associated variables is remarkable. Moreover, partial correlation analysis performed to remove interference from other variables (RBC, Hb, PLT, splenomegaly, and Child-Pugh class) confirmed the relationship. This association between varix severity and RBC lifespan could be explained, at least in part, by the fact that although hypersplenism may lead to increased blood cell destruction and hemocytopenia, peripheral blood cell metrics reflect a balance between consumption and hematopoiesis. Furthermore, whereas WBC concentrations in circulation are regulated heavily by mobilization mechanisms, RBC lifespan depends only on the rate of destruction of circulating RBCs and is not affected by other factors, such as bone marrow compensation.

Although prediction of VNT presence with Baveno VI criteria (liver stiffness and PLT components) is highly sensitive, it has only mediocre specificity, leading to unnecessary EGD in large numbers of patients.^6,10,11^ Only PLT was evaluated in this study. Consistent with previous reports, thrombocytopenia based on PLT was detected in substantial portions of patients without severe varices. If a liver stiffness test had been applied as well, it likely would have yielded additional false positives. The infrequency of false positives obtained based on our RBC lifespan metric (a single patient with mild varix severity) suggests that the prediction specificity of RBC lifespan should be much higher than that obtained based on standard PLT cutoffs. Conversely, because there were no patients with severe esophageal varices who had a false negative based on RBC lifespan, the prediction sensitivity of RBC lifespan measurement would not be expected to be lower than that based on thrombocytopenia alone. The VNT prediction performance of RBC lifespan may thus be further improved by combining an RBC lifespan criterion with a transient elastography criterion in a combination rubric.

The major limitations of this study are its small sample size, particularly of patients with mild and moderate varices, and the lack of a comparison with transient elastography outcomes. The LCOBT itself also has some limitations. Firstly, smokers are not well suited to breath testing due to tobacco-associated increases in CO release. Substantial numbers of patients with cirrhosis are smokers, especially among those with alcoholic cirrhosis. For this group of people, quantitative liver function tests, especially those that have been evaluated in the famous HALT-C trial, might be considered. For example, the oral cholate clearance test was found to have a sensitivity as high as 86% for predicting the severe clinical outcomes.^24,25^ Secondly, like the classic ^51^Cr-labeling method of measuring RBC lifetime, LCOBT is highly sensitive and specific for detecting hemolysis, but does not provide information about the underlying cause of hemolysis (eg, hypersplenism or not) and cannot differentiate between intravascular hemolysis and extravascular/in situ hemolysis (i.e., ineffective hematopoiesis). Definitive diagnosis requires clarification of the etiology and location of hemolysis. These limitations, however, do not affect the basic conclusion that there is a highly significant inverse correlation between RBC lifespan and VNT presence in cirrhotic patients.

## CONCLUSION

RBC lifespan shortening in patients with cirrhosis worsens with greater severity of esophageal varices. The simple and rapid LCOBT has the potential to be used as a tool to predict which patients are likely to have VNT. The present findings should be validated in a large-sample study.
